# Editorial: Occupational exposure to nanomaterials

**DOI:** 10.3389/ftox.2022.1014600

**Published:** 2022-09-20

**Authors:** Charlene Andraos, Mary Gulumian, Gaku Ichihara, Boowook Kim, Il Je Yu

**Affiliations:** ^1^ National Institute for Occupational Health (NIOH), National Health Laboratory Service (NHLS), Johannesburg, South Africa; ^2^ Water Research Group, North West University, Potchefstroom, South Africa; ^3^ Department of Occupational and Environmental Health, Faculty of Pharmaceutical Sciences, Tokyo University of Science, Tokyo, Japan; ^4^ Institute of Health and Environment, Seoul National University, Seoul, South Korea; ^5^ HCT America, Morgan Hill, CA, United States

**Keywords:** nanomaterial, occupational, exposure, nanoparticle, toxicology

Nanotechnology by definition is the “manipulation of matter on a near-atomic scale to produce new structures, materials and devices” to promote “scientific advancement in medicine, consumer products, energy, materials and manufacturing” (NIOSH, 2020a). The release of nanomaterials (NMs) from these products, whether at production stage, use or during disposal, appears to be inevitable thereby exposing workers, consumers, and the environment to potentially hazardous materials. Despite the ever increasing use of, and exposure to, NMs, their exposure assessment in occupational settings is challenging at best. The assessment is hindered particularly by factors identified in a systematic review conducted by Ghafari et al. (2020). For example, 1) quantitative information about NM characteristics and exposure scenarios in occupational environments are scarce and unreliable; 2) due to a lack of consensus among researchers regarding the correct quantitative methods and equipment to use for assessment of NM exposure it often results in an unfocused approach where a multitude of equipment and techniques are being employed; 3) there is also no consensus on the most appropriate metric to be used to assess exposure. Although surface area concentration is regarded to be the most appropriate, widespread adoption of this metric appears to be slow; 4) the current recommended occupational exposure limits (OELs) for limited number of NMs may not effective enough. Therefore, additional/alternative exposure metrics such as inhalable and respirable and total nanostructures and possible outcomes regarding health, oxidative stress and inflammation and long term-effects should be considered when establishing/revising an OEL; 5) The biomarkers examined to date do not have the necessary sensitivity and specificity for NMs. It is therefore suggested that more sensitive and specific biomarkers be identified to assess occupational exposure to NMs. It should also be noted that the current emergence of advanced materials and micro/nano plastics may also present with some of the same challenges as seen with NMs. For example, advanced materials and micro/nano plastics may also suffer from the lack of ideal detection and quantification techniques.

These challenges mentioned above prompted the establishment of a Research Topic entitled: “*Occupational Exposure to Nanomaterials*” and is an all-encompassing edition of the challenges faced by NMs as well as the more recent micro/nano plastics and 3D printing emissions. Indeed, exposure to these materials are faced by many in the occupational realm and needs to be highlighted. Four, high-quality and highly relevant articles, i.e., one review and three original research manuscripts, have been published in this Research Topic.

The aim of the review by Mariano et al., entitled “*Micro and Nanoplastics Identification: Classic Methods and Innovative Detection Techniques* ” was to address the most suitable microscopical, analytical, and chemical characterization methods for the detection, characterization and identification of micro/nano plastics in different matrices. They further discuss the challenges to enhance these existing methods and the potential to develop new methods. Inevitably, microscopic techniques are more often than not coupled with analytical techniques to aid in identification and chemical characterisation. For example, an FT-IR or Raman spectroscope equipped with a microscope has generally been used for the chemical identification of polymers at the microscale. In high demand of course, is the implementation of novel techniques that may overcome the drawbacks of existing methods. One of these novel techniques, coined “digital holography (DH)”, allow for the identification of a large number of plastics in complex matrices containing several other organic and inorganic structures/pollutants with similar sizes and shapes as that of plastics. DH involves the use of artificial intelligence and a holographic microscope sensor. Visual information is stored with the use of lasers whose holographic registration is carried out through the sensor. The artificial intelligence system is then trained to distinguish plastics from other natural materials.

The adverse health effects from exposure to emissions released from 3D printing has also recently been highlighted (NIOSH, 2020b). 3D printing involves the use of a digital file to build a physical 3D object by successively adding layers of material or filament until the final product is complete, i.e., in short, it is “additive manufacturing”, as opposed to traditional manufacturing methods in which a block of material is sculpted into the final product (OECD, 2017). The process of 3D printing has shown to emit several hazardous pollutants such as micro- or nano-sized particles and volatile organic chemicals (VOCs), depending on the type of filament used. In the study by Kim et al., entitled “*Assessment and Mitigation of Exposure of 3D Printer Emissions*”, a workplace equipped with two 3D printers using acrylonitrile-butadiene-styrene (ABS) copolymer filaments was assessed. The importance of engineering controls for the mitigation of exposure was stressed in this study as exposure to 3D printer emissions was greatly reduced after isolating the 3D printers in enclosed spaces. The same authors then went a step further and introduced the use of a cell-based biomonitoring device that can evaluate acute cytotoxicity and effect biomarkers such as inflammation at the same 3D printing facility. Their published research Kim et al., entitled, “*On-Site Deployment of an Air-Liquid-Interphase Device to Assess Health Hazard Potency of Airborne Workplace Contaminants: The Case of 3D Printers*”, involves the exposure of A549 lung epithelial cells at the air–liquid interphase (ALI) to workplace air for 1–2 h. Interestingly, the mRNA expression of pro-inflammatory cytokine IL-1b and IL-6 increased significantly after 2-h exposure to 3D printer emissions while IL-6 continued to increase after 24-h. In contrast, the expression of TNF-α mRNA decreased significantly after 2 h of exposure to 3D printers and decreased further after 24-h post-exposure. ALI devices are indeed showing great promise in real-world exposure scenarios and could aid in the biomonitoring and risk assessment of air pollutants.

Our last featured article from Masekameni et al., “*Exposure Assessment of Silver and Gold Nanoparticles Generated During the Synthesis Process in a South African Research Laboratory*” stressed the importance of modelling deposition patterns of metal-based NMs, which could provide further insight into their possible adverse health risks. For the three exposure scenarios assessed at the research laboratory in South Africa, none exceeded the occupational exposure limit for both AuNPs (provisional: 20,000 particles/cm^3^) and AgNPs (OEL: 0.19 μg/m^3^) although the concentrations to which laboratory workers were exposed indicate a high particle lung retention, based on computational assessment using the Multiple Path Particle Dosimetry (MPPD) Model, which raise concerns about the long term safety of workers. Therefore, together with the OEL, other factors should also be taken into account that may provide a more realistic health prediction outcome and which may also allow re-evaluation of current OELs.

In conclusion, this edition is an excellent representation of the recent studies addressing the challenges faced by exposure assessment of NMs, as mentioned previously. For example, Mariano et al. addressed the challenges faced with detection of micro/nano plastics in different matrices using current methods and also introduces novel detection techniques. Kim et al. on the other hand showed the successful mitigation of 3D printer emissions (Kim et al.) whilst addressing the lack of current sensitive and specific biomarkers by introducing an *in vitro* ALI-based biomonitoring device (Kim et al.). Lastly, Masekameni et al. stresses the need to not just accept current OELs at face value but to rather incorporate additional computational approaches that may provide more information on the long term health effects of NMs that may in the end assist with OEL revisions. The editors wholeheartedly thank each of the authors who contributed to the success of this Research Topic.
